# Gaining More Ease in Everyday Life as a Family With a Child With Intellectual Disability Through Family-Systemic Therapeutic Conversations: An Exploratory Single-Case Study

**DOI:** 10.1177/10748407251392885

**Published:** 2025-11-14

**Authors:** Simone Pascale Wildhaber, Corina Sgier, Margrit Hilpertshauser, Evelyn Huber

**Affiliations:** 1 ZHAW Zurich University of Applied Sciences, Winterthur, Switzerland

**Keywords:** family systems care, family health, therapeutic conversations, intellectual disability

## Abstract

This study aimed to learn how a sequence of family-systemic therapeutic conversations created a context to respond to the challenges and needs of a family raising a child with intellectual disability. The data for this qualitative, exploratory, longitudinal single-case study were collected at a Swiss Family Systems Care Unit. The three therapeutic conversations conducted with a single mother of an adolescent with intellectual disability were analyzed using summarizing and explicative content analysis on the within-data source level and structuring content analysis on the across-and-between data source level. The main category, “gaining more ease by modeling burdens and suffering,” refers to a better balance of the woman’s challenges cumulating to deep suffering. Six subcategories detail the challenges, needs, interventions, and effects. Therapeutic conversations in families with multiple severe vulnerabilities are supportive. The study was written according to the Standards of Reporting Qualitative Research and the Methodological Framework for Organizational Case Studies.

## Introduction

### Background

Worldwide, 3.4% of all children and adolescents under the age of 20 are affected by an intellectual disability (Cieza et al., 2021). In economically stable countries, this prevalence is 2% to 3% (Totsika et al., 2022). In Switzerland, according to the Federal Statistical Office (2019) in 2017, around 5% (approx. 54,000) of all children between the ages of 0 and 14 lived with a disability, with 1% (approx. 10,000) of all children affected to a severe degree. 16% of the children with disability had an intellectual disability with significant consequences for those affected and their families. Children and adolescents with intellectual disabilities are more likely to have physical and psychiatric comorbidities and behavioral problems than those without intellectual disabilities (Totsika et al., 2022). At the same time, they have a higher risk of inadequate treatment (Haller et al., 2022; Totsika et al., 2022).

Parents of children and adolescents with disabilities have more stress (Baker et al., 2021), mental health problems, depression (Marquis et al., 2020; Smith & Grzywacz, 2014), chronic physical illnesses, and an unhealthier lifestyle compared with parents of children without disabilities (Lee et al., 2017). Many suffer from chronic sorrow due to feelings of guilt or because their children miss out on expected developmental milestones, have limited opportunities for participation, and an uncertain future (Coughlin & Sethares, 2017). Mothers appear to be more affected by chronic sorrow and mental health symptoms than fathers (Coughlin & Sethares, 2017; Marquis et al., 2020). In addition, parents are confronted with loss of income and additional costs due to their child’s disability (Mitterer et al., 2021; Roddy, 2022; Wondemu et al., 2022). They are more affected by poverty, less likely to be employed, and less likely to have private health insurance (Lee et al., 2017). Mothers have more income losses and reduced workloads than fathers (Wondemu et al., 2022).

A longitudinal, 10-year study showed that feeling more in control of the situation supported the mental and physical health of affected parents and protected them from depression, mothers more than fathers (Smith & Grzywacz, 2014). Practical support from outside the family promoted mental health (Smith & Grzywacz, 2014). Satisfaction with the support received and active coping strategies reduced stress and increased the sense of gain from care work (Grey et al., 2018). In terms of support from health care professionals, empathy and compassion, information provision, partnership, and respite options were described as particularly helpful by both parents and health care professionals (Coughlin & Sethares, 2017; Pilapil et al., 2017). However, access to health care professionals able to deal with the complexity of the situations of affected families is limited (Totsika et al., 2022).

One intervention in advanced nursing practice to support families in complex health-related situations is family-systemic therapeutic conversations (Shajani & Snell, 2023). Such conversations aim to strengthen families’ symptom and everyday life management, soften their suffering, and promote or restore their health and well-being (Wright & Bell, 2021). Therapeutic conversations are based on the Calgary Family Assessment Model, the Calgary Family Intervention Model (Shajani & Snell, 2023), and the Illness Beliefs Model (Wright & Bell, 2021). The Calgary Family Assessment Model provides a structure to assess and understand complex family situations in the dimensions of “structure,” “development,” and “function” (Shajani & Snell, 2023). The Calgary Family Intervention Model describes interventions by health care professionals to promote, improve, and maintain family functioning, meaning how family members behave regarding family routines and communication, in the domains of “cognition,” “affect,” and “behavior” (Shajani & Snell, 2023). According to the Illness Beliefs Model of Wright and Bell (2021), health and illness are understood in relation to the physical, emotional, relational, and/or spiritual domains. Beliefs of families about health and illness in these domains are the basis for coping with health and illness issues. Change and healing in families can happen, when facilitating beliefs are strengthened and constraining ones challenged.

In pediatric populations, therapeutic conversations were mostly investigated in Iceland (Foster & Shields, 2020) using quantitative study designs. In family caregivers of children and adolescents with various physical or mental illnesses, therapeutic conversations improved family functioning, illness beliefs, perceived family support, satisfaction with health care, and quality of life, particularly in mothers (Gisladottir et al., 2017; Ragnarsdóttir & Svavarsdottir, 2014; Svavarsdottir et al., 2014, 2020; Svavarsdottir & Gisladottir, 2019; Svavarsdottir & Sigurdardottir, 2013). According to a grounded theory study with five families experiencing chronic illnesses of a child or a parent, a few therapeutic conversations helped the mothers to better balance the multiple burdens that had almost broken them and made it easier for the mothers to deal with their suffering (Robinson, 1998).

There is a need for more qualitative studies to understand more in depth how therapeutic conversations support families with ill children and adolescents. Moreover, Foster and Shields (2020) identified the need to connect clinical practice and research of therapeutic conversations in pediatric populations. Although family systems care has become established with positive outcomes for children with disabilities and their families, for example, in the early intervention sector (McCarthy & Guerin, 2022) or for families with a child with cerebral palsy (Hayles et al., 2015), our literature search in the databases PubMed, CINAHL, Medline, Carelit, and Google Scholar revealed that little research has been done on the health-related challenges and needs of families with children with intellectual disabilities, as well as the benefits of therapeutic conversations in this population.

### Aim and Research Questions

The first aim of this study was to gain an in-depth understanding of the health-related challenges and needs faced by one exemplary family with a school-age child with intellectual disability. We focused on school-age, since we expected the family to have long-term experiences with complex health-related challenges and needs. In the context of this exploratory study, we did not restrict the type of intellectual disability because diagnoses are often lacking or rare.

The second aim was to understand in what way family-systemic therapeutic conversations created a context, in which this family felt recognized in their needs and supported in managing the effects of the families’ challenges.

The following research questions were investigated: “What health-related challenges and needs does a family with a school-age child with intellectual disability describe in the context of therapeutic conversations?” and “How can the family be supported in dealing with their challenges in the course of three therapeutic conversations?”

## Method

### Research Design, Casing, and Setting

A qualitative, exploratory, longitudinal, embedded single-case study design was used (Yin, 2018). Case study designs are suitable for investigating families as complex systems comprehensively (Schulz et al., 2021). The individual case serves as an illustration of a phenomenon in a specific context (Creswell & Poth, 2018; Yin, 2018). In a bounded system, a case is limited by place, time, and activity (Sandelowski, 2011; Yin, 2018). Information is collected broadly to describe the case in a multifaceted way and to be able to learn from it for similar situations (Creswell & Poth, 2018; Yin, 2018). In the embedded case study design, a single case comprises several units of analysis (Yin, 2018).

In this case study, a family with a school-age child with an intellectual disability was defined as the case and included in the study. The case was bounded in terms of location by the premises of the Family Systems Care Unit of a Swiss university of applied sciences, in terms of time by a sequence of three therapeutic conversation sessions, and in terms of activity by the therapeutic conversations according to the Calgary Family Assessment and Intervention Models (Shajani & Snell, 2023) and the Illness Beliefs Model (Wright & Bell, 2021).

The Family Systems Care Unit offers therapeutic conversations for families or individuals with burdening health and illness issues by an interprofessional clinical team of health care professionals. Most of the clinicians are advanced practice nurses trained on a Master of Science level and specialized in family systems care and family-systemic counseling. The Family Systems Care Unit premises comprise a counseling room and an adjoining room with a one-way window in between. This infrastructure allows members of the clinical team as well as researchers and students to observe the therapeutic conversations in the adjoining room. The therapeutic conversations at the Family Systems Care Unit are free of charge. Further information on the Family Systems Care Unit can be found in Huber et al. (2024).

### Recruitment

For recruitment, the last author contacted one organization in the social sector working with families of school-age children between 4 and 18 years and explained the study to two leaders of this organization. Based on a purposeful sampling strategy (Creswell & Poth, 2018), the leaders were asked to approach a small number of families regarding their interest in participating in this study in connection with a series of single-case studies, which were then to be compared with each other in a cross-case study (Huber et al., 2024). Inclusion criteria were families raising a child with intellectual disability and willing to share their health-related challenges and needs in the premises and under the conditions of the Family Systems Care Unit. Families with poor or no knowledge of German were excluded from the study.

The two leaders contacted three families who confirmed their interest in participating in this study and agreed that the leaders of the organization provided their telephone numbers to the study team. The head of the Family Systems Care Unit contacted them by telephone and, after explaining the study, arranged appointments for the therapeutic conversations. One of these three families forms the case for this study.

### Data Collection

The data were collected from therapeutic conversations at the Family Systems Care Unit. The family, in this case study a woman who is a single mother of a 12-year-old son with intellectual disability and a 9-year-old daughter without disability, was invited to participate in three therapeutic conversations between November 2022 and January 2023, with the option of attending further ones if required.

In these conversations, the woman was asked to tell the counselor, an experienced advanced practice nurse and family systemic counselor, about her and her family’s experiences and health-related challenges in the context of raising a child with intellectual disability. The counselor started the first conversation by asking: “Could tell me about your situation? What does your everyday life look like? Who is part of it?” While the woman started to describe her family situation, the counselor begun to draw a genogram-ecomap. The genogram is a visualization tool in accordance to the structural family assessment to envision the family structure (Shajani & Snell, 2023). The ecomap supplements the genogram by visualizing the family’s contacts beyond the inner family members (Shajani & Snell, 2023). The counselor deepened the conversations by validating and acknowledging the woman’s experiences, asking questions, and providing information relying on the Calgary Family Assessment and Intervention Models (Shajani & Snell, 2023) and the Illness Beliefs Model (Wright & Bell, 2021). Examples of such questions or statements were: “What are you and your children concerned about at the moment?,” “Is there anything you need an explanation or a tip about?,” or “The fact that you organize yourself and go there is a huge achievement.”

Three therapeutic conversation units formed the foundation for this study, each consisting of the following components: (a) a preliminary session in the clinical and research team, (b) the therapeutic conversation with the woman, and (c) the evaluating session of the clinical and research team. In this study, the counselor, one or two other members of the Family Systems Care Unit clinical team, the first author, and the last author were present in the preliminary and evaluating sessions. The preliminary sessions (a) lasted between 45 and 70 minutes. The team discussed possible relevant content for the therapeutic conversation based on information already available. The therapeutic conversations (b) with the woman lasted between 60 and 80 min. In 45- to 70-min evaluating sessions (c), the content of the therapeutic conversations was reflected on by the team. All sessions were recorded on video or audio. The data material comprised a total of 610 min. Notes were taken during all conversations by the first author and themes were identified to be able to observe them specifically in the next therapeutic conversation unit.

### Data Analysis

For the analysis of the data, the audio recordings were transcribed verbatim by the first author. The transcripts were organized using *MAXQDA 2022 (VERBI Software, 2021)*. The video recordings were used to supplement the analysis.

**Figure 1.**
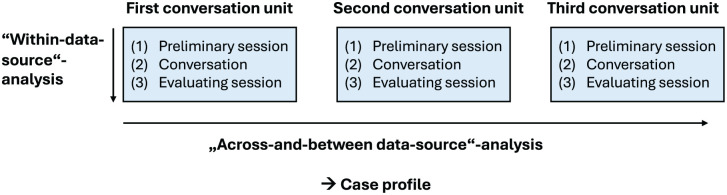
Schematic Representation of the Data Collection and Analysis Sequences Based on Sandelowski’s (2011) Method.

Following Sandelowski (2011), the data were first analyzed on the “within-data-source” level and then on the “across-and-between-data-source” level. The procedure is visualized in Figure 1. The data were analyzed using content analysis (Mayring, 2015).

### Within-Data-Source Analysis

On the within-data-source level, the three conversation units, that is the conversations with the respective preliminary and evaluating team sessions, were analyzed in themselves (Sandelowski, 2011).

Following Mayring (2015), an inductive summarizing content analysis was used to condense the content regarding the family’s challenges and needs in six iterative abstraction steps. First, conversation passages that were relevant to the first research question were paraphrased. These paraphrases were then converted into more abstract descriptive codes. In the following two steps, descriptive codes with similar meanings were combined and further abstracted and thus reduced, by first grouping together similar descriptive codes and then by further bundling and relating grouped codes into more abstract categories. Finally, the abstracted categories were reviewed with the source material and more refined as needed.

In a further iterative step, still following Mayring (2015), the interventions the counselor used in the therapeutic conversations, such as the questions asked or the commendations or explanations expressed, and the woman’s reactions to them with regards to her previously identified challenges and needs were explored in depth in an explicative content analysis using a narrow and an extended context analysis of the conversations. That is, the content of the conversations close to an intervention was explored in depth by including the video and audio recordings in addition to the transcripts. Progressions of the interventions and the woman’s reactions were then identified in an extended context analysis looking at the progression during an entire conversation session. In an additional deductive analysis step, the interventions and the woman’s reactions to them were explored regarding the family functions domains “cognition,” “affection,” and “behavior”, according to the Calgary Family Intervention Model (Shajani & Snell, 2023), to gain further insights into the effects of the conversations.

### Across-and-Between Source Analysis

Subsequently, the three conversations and the respective preliminary and evaluating sessions were compared with each other on the “across-and-between-data-source” level (Sandelowski, 2011). Based on the results of the “within-data” analysis, the challenges, needs, interventions, and reactions were related and compared looking at the process between the conversation units. For this, we relied on Mayring’s (2015) structuring content analysis and compared the insights of the “within-data” analysis in relation to the “cognitive,” “affective,” and “behavioral” family function domains (Shajani & Snell, 2023) to further condense the content regarding the research questions. Preliminary categories and subcategories were then named, defined, and supported with anchor examples from the source data before reviewing them on the source data until the final set of categories and subcategories was developed (Mayring, 2015).

### Ethical Considerations and Data Management

According to the Cantonal Ethics Committee of Zurich, this study was not subject to the Swiss Human Research Act (Req-2021-01424). The present work was conducted in accordance with the principles of the Helsinki and thus complies with the ethical principles for medical research involving human subjects (World Medical Association, 2013). Following these principles, the woman was informed about the study verbally and in writing by the counselor moderating the conversations and signed an inform consent form before participating. Participation was voluntary. All those present in the preliminary and evaluating sessions were also informed about the study and signed an informed consent form and a confidentiality agreement form regarding the handling of the data in this study. The participants were informed of their right to withdraw from participation at any time and to object to the use of the audio and video recordings and other data after the therapeutic conversations. The data was stored in a secure folder on a server at the university of applied sciences, to which only people who work directly with the data clinically or for research purposes have access. The transcripts and consent forms are stored in a secure archive by the Family Systems Care Unit management for 10 years (electronic data: password protected, paperwork in a secure archive at the university of applied sciences).

### Quality Criteria

Six quality criteria of qualitative research according to Mayring (2016) were used for this study. “Process documentation” was achieved by documenting all analysis steps in a shared folder. “Argumentative validation of the interpretations” took place by reflecting the progresses of the data analysis with the team of clinical experts and researchers. “Compliance with the rules” was ensured by critically scrutinizing the analysis plan in consultation of the first author with the last author. “Closeness to the subject” was maintained by examining a real therapeutic conversation situation of the everyday world of the participating woman. “Communicative validation” was achieved by reviewing the results together with the counselor who moderated the therapeutic conversations. “Triangulation” was followed by analyzing the data from three data collection points and the data from the therapeutic conversions, preliminary and evaluating sessions.

## Results

### Family Situation

The family is composed of a woman, her 12-year-old son, and 9-year-old daughter. The woman’s mother who lives nearby supports the nuclear family in everyday life. The children are in contact with their father, however, the relationship between the parents is strained. The son is affected by intellectual delay diagnosed at the age of 3. He has limited speech abilities and needs more support in social interactions and daily activities than other children of his age. He has motor function impairments, still is fully mobile. The woman grew up outside Switzerland, speaks fluent German and completed a vocational training in health care in Switzerland. Since birth, she has suffered from a disabling physical health problem with currently pronounced symptoms that has caused inability to professional work.

### Main Categories and Subcategories

A main category with six subcategories was identified from the analysis. The main category “gaining more ease by modeling burdens and suffering” points to the overarching impact of the therapeutic conversations on how the family found support in dealing with its various challenges and needs arising from severe burdens and cumulating to deep suffering. The subcategories detail the burdening challenges and suffering, the ensuing needs related to health, the interventions offered during the therapeutic conversations as well as the effects that were reported by and could be observed in the woman.

#### Main Category: Gaining More Ease by Modeling Burdens and Suffering

“Gaining more ease by modeling burdens and suffering” describes the overall impact of the therapeutic conversations for the woman and her family regarding her and her family’s health-related challenges that revealed as severe burdens causing deep suffering in this woman. From the outside, the woman made a strong and self-confident impression. The counselor asked whether this impression corresponded with her emotional world. The woman replied,

It’s not what it actually looks like from the outside . . . I’m depressed, sad . . . I just cry a lot, I don’t know what the reason is . . . (Woman, conversation 1, position 271)

The main category, as an overall impact of the therapeutic conversations, indicates to a better balance in coping with the different burdening challenges and related needs and thus to relieved suffering. “Modeling” the woman’s burdens and suffering by talking and reflecting on them in the conversations led to a feeling of more ease and balance translating into her family life. By sharing her experiences and concerns in the therapeutic conversations, the woman took advantage of the conversations to make her and her family’s health-related physical, emotional, interpersonal, cultural, resource-related, and organizational burdens more bearable and to cope with them more effectively. The burdens and suffering were not resolved but made conscious with new perspectives on them being developed. The conversations helped the woman to find new ways of dealing with her situation. At the end of a therapeutic conversation, she was asked how the conversation had been for her, to which she responded:

Good, relieving . . . one really can’t talk so openly with every person . . . You give confidence that one feels comfortable. (Woman, conversation 1, position 469)

By telling the personal story, the woman could express her suffering. One of the clinical experts commented on this in a pre-session:

. . . permission for suffering and to talk about suffering and listening to suffering is the first step to healing. (Clinical expert, pre-session 3.1, position 15)

The proceeding of this healing process was evident in the following subcategories.

#### Subcategory 1: Fighting for the Own Health for Oneself and the Children

This subcategory refers to the process by which the woman makes an intensive and persistent effort to take care of her own health despite many challenges, and thus to be able to look after both herself and her children.

The woman identified her own health as a priority issue:

“You can’t do without health . . .” (woman, conversation 2, position 149). “. . . I must look after myself and I am important. I come first and then the others” (woman, conversation 1, position 411). “If it works with me, then it also works with the children” (woman, conversation 1, position 413).

The care and raising of her son, which places great demands on the woman, exacerbates her own physical and mental symptoms:

. . . He’s a special child, he’s, he really takes a lot of energy from me because he’s not like twelve, he’s like six or even five. And yeah, you always must take your time . . . fight with him to make him do something . . . he just does things the way he wants, he has a mind of his own . . . (Woman, conversation 1, position 55)

The woman emphasized fighting for her own health as a challenge and a need. Improving and stabilizing her state of health is of great importance for the further care of the children from the woman’s point of view. She has started ambitious sporting activities to reduce her physical pain and chronic fatigue. However, finding time for these activities in her busy everyday life is a challenge.

The counselor recognized and appreciated the woman’s efforts and was responsive by providing specialist information. During the conversations, it became clear how excessive training intensity can evoke negative emotions, which in turn lead to an increase in pain and fatigue. The counselor engaged in discussing the importance of setting realistic goals and gradually increasing sporting activity. The woman said in a conversation about her goals in sport:

I’ve always set myself a goal that I can’t even achieve . . . because it’s too difficult. (Woman, conversation 2, position 221)

The counselor initiated discussing more realistic goals that would help the woman to find a balance between training and relaxation. She suggested to the woman that she should not have to do even more and emphasized the importance of recovery phases. The woman confirmed this in the conversation, expressed that she felt understood, adjusted her training and activity plan, and set herself adapted goals to achieve a better health status.

#### Subcategory 2: Finding Peace as a Family and as an Individual

This subcategory expresses how the woman strives for a healthy environment for recreation and family development for herself and her family and wants to create space for balancing the different needs of her children and her own.

The need for peace and quiet was expressed by the woman like this:

. . . Finding peace, inner peace also at home. (Woman, conversation 1, position 403)

The family was shaken by severe strokes of fate before and after the birth of the children and is now in the process of establishing calm in the home environment. After the difficult separation from her husband, the woman has gained a sense of ease and thus created a family home where all of them feel well, and their needs can be met. This supports relaxation after difficult times and restructuring of the family life:

. . . The children are really having a great time and are happy. Yes, that makes me happy, so I’m proud that I’ve managed to get them to enjoy being at home and they just want to be at home all the time. Yes, that’s something that’s not a given everywhere. (Woman, conversation 1, position 401)

The creation of a genogram-ecomap provided insights into the complexity of the family’s issues. Through the counselor’s questions and modeling in the genogram-ecomap, the woman explored the family’s values, beliefs, relationships, perspectives, and needs and was thus able to gain new insights.

In everyday life, the woman is constantly faced with the challenge of finding a balance to meet the needs of all family members. Looking after her son is very time-consuming and takes up a lot of the woman’s resources and energy, which causes reactions in her daughter. The woman described the relationship between her children this way:

She fights back, she’s angry, but she loves him anyway, unconditionally, she’d do anything for him, she’s like a mother to him sometimes, really. When I’m not there, she supports him. But when I’m there, she probably wants me to be more of a mommy for herself. (Woman, conversation 1, position 75)

The counselor highlighted and valued the woman’s strengths, such as the woman’s thoughtful recognition of the children’s relationship and need for space for each of them. The woman was open to discuss ideas for action, such as planning times with the daughter if the grandmother would look after the son. Various options of this kind could help to better balance the resources of the family members and create personal space and a new togetherness for everyone.

#### Subcategory 3: Liberating From Feelings of Guilt and Blame

This subcategory describes the process in which the woman works to relieve herself from burdening feelings of guilt and blame, which is closely related to her need for inner peace. This process includes the adequate integration of negative feelings into her life story and the challenge and attempt to create a positive self-image.

The family’s story is characterized by various questions of guilt that add to the woman’s suffering and contribute to burden her health. There are feelings of guilt by the woman herself and external blame. Accepting her son’s disability was a considerable challenge:

It took me a long time to accept the disability, because back then, when I talked about him, I was in tears. I felt bad, I couldn’t talk about him being like that. But now I can say: yes, he is like that. (Woman, conversation 1, position 337)

Her husband’s coping strategy, who kept struggling to accept their son’s disability and saw the mother’s upbringing as the reason for his developmental delay, additionally burdened the woman:

The husband just accused me the whole time and also just dragged me down with the conversations, with the accusations. (Woman, conversation 1, position 219)

The couple’s dynamic resulted in deterioration of the woman’s physical and mental symptoms threatening her ability to take care of her son’s and daughter’s needs.

The use of financial support also triggered feelings of guilt, which was reinforced by negative experiences when visiting the authorities. The woman expressed high expectations of herself, and the feelings of guilt led to her wanting to achieve even more.

The counselor emphasized the valuable service the woman provides to society as a mother raising her children and expressed her commending and appreciation for the woman’s strengths and competences in being a mother. Using the method of “reframing” encouraged the woman to transform stressful thought patterns into friendlier ones and thereby addressed the need to take pressure from herself:

Now you have to start labeling this nap time differently. This is “healing sleep,” “recovery sleep,” this is a “cure” that you are taking. (Counselor, conversation 1, position 462)

This reinterpretation gave rather negative aspects, which were associated with feelings of guilt and blame, a new healing meaning as “moments of relaxation and regeneration.”

#### Subcategory 4: Belonging Despite Feeling Foreign

This subcategory points to the challenge that the woman often feels foreign in Switzerland due to her cultural background and political views. This includes the ambivalence between distancing and silence and the need for connection, sharing her history, and raising awareness of political unfairness.

The complex cultural background of the family is associated with various health-related challenges. Although the family is integrated and feels safe, the woman’s subjective well-being is impaired. She mentioned her emotional distress in this regard and her concern that her children will never really belong here. Throughout the therapeutic conversations, the woman’s great emotional suffering due to political injustices became apparent:

At the end of the day, we are all human beings, and we come into the world without clothes, without language, without religion, without anything. Right? And then the only difference is where we are born, and we can’t choose that. Nobody can choose that. And why are we ruining everything because of this? (Woman, conversation 1, position 115)

The woman reported that she felt a great necessity to tell everyone about these injustices, to make people aware of them, but on the other hand she had the impression that talking about them made her feel sad and that she could not change anything. She comments on this like that:

I’ve been talking a lot lately. My voice has gone. Yes . . . it’s no use talking so much. Yes, I just want to, I would have loved to tell everyone what happens there. (Woman, conversation 3, position 231)

The counselor acknowledged the suffering due to political injustices and validated the negative feelings related to the family’s cultural suffering.

The woman also attributed some of her parenting challenges to her cultural background. The counselor shared her own experiences of parenting and normalized the children’s behavior. She emphasized that Swiss children follow the same patterns and that the experiences of the family are not limited to their cultural background, but that many families have similar challenges in raising their children.

#### Subcategory 5: Desiring Independence and Accepting Help

This subcategory describes the great challenge to maintain independence while also needing to accept help. This includes the realization that not everything can always be managed alone, and that accepting support can be relieving. The woman assumes that her son will not achieve an autonomous and independent life and depend on ongoing support in every day live.

The family is also currently dependent on financial support. The financial worries are always present and have a negative impact on the family’s social life and the woman’s health:

. . . We always must watch every centime . . . (Woman, conversation 2, position 105)

The woman mentioned that there are friends and relatives in her environment who could offer support, but she is reluctant to accept this help. She has not yet taken advantage of external support options. The organizational effort involved in taking advantage of respite services was causing the woman stress and tension. Also, she did not want to burden her friends and relatives.

The counselor adopted a commending attitude toward the woman and recognized her courage and strengths in coping with the complex demands of single parenthood after the separation:

So, your children can be proud of their mother. (Counselor, conversation 1, position 364)

Throughout the conversations, the counselor provided information about various relief options without giving the feeling that the woman had to make use of them. Support from the counselor in organizing these offers could create space for more ease. The woman responded that she would generally like to take advantage of respite services, also to provide her son with a social environment in this context.

#### Subcategory 6: Finding Meaning or not Needing to Find Meaning

This subcategory is about the perspective that it is not always necessary to look for a deeper meaning in everything. It is more helpful to focus on fulfilling needs and finding meaning and joy in small moments.

Accepting the son’s disability created suffering in the woman. When she was able to accept her son as he was, she was able to stop looking for a meaning or reason for his disability, which made it easier for her.

In one of the conversations, the woman was very emotionally affected by recent events. The woman referred to this during the conversation:

. . . I also always ask myself about the meaning of life, but I can’t find an answer. (Woman, conversation 3, position 165)

The counselor took up this question:

Do you think there is an answer? (Counselor, conversation 3, position 166)

The woman replied:

I don’t think there is just one. (Woman, conversation 3, position 167)

The counselor did not respond verbally to the woman’s answer. Through the relationship and confidence that has been established by then between the counselor and the woman, the unspoken was continued on a different level in a moment of silence. The result was a form of communication that went beyond the verbal. This facilitated the woman to relax both mentally and physically.

## Discussion

The family in this case study was confronted with several severe burdening challenges that contributed to the woman’s suffering. The therapeutic conversations provided a setting, in which the woman started modeling these burdens and suffering, which helped the woman to gain more ease. These conversations further allowed to explore some of the woman’s and her family’s needs that emerged throughout the conversations or were addressed directly by the woman. The challenges and needs posed by the son’s intellectual disability, which formed the focus of this study, proved to be one of numerous issues that burdened the mother and, in sum, caused her deep suffering (Wright, 2017). Among other burdens, the woman herself was affected by an innate serious disabling health condition. Thus, this case study exemplifies the importance of considering the various challenges and needs of families in all their complexities (Huber et al., 2021).

The therapeutic conversations offered the woman a space to tell her and her family’s story in its complexity. Facilitating narratives about one’s experiences is one of the most common interventions of family-systems therapeutic conversations (Azcárate-Cenoz et al., 2024). This legitimizes feelings related to distress and suffering and recognizes the strengths in dealing with them (Duhamel, 2021; Shajani & Snell, 2023). Reconstructing one’s own narrative can help to strengthen facilitating beliefs in dealing with one’s history, change constraining ones, and thereby soften suffering and supporting the affective family function domain (Wright & Bell, 2021). Meaning and sense are created in narratives (Wright & Bell, 2021). Listening to families’ narratives in a non-judgmental way and legitimizing emotional reactions enables health care professionals not only to enter into partnership with families, but also to develop new, shared perspectives on health and illness and create a space for change (Duhamel, 2021; Murphy & Franz, 2019). Even in short therapeutic conversations of 30 min on average with parents of children with rare diseases, listening to narratives was used as an essential element, which increased the positive perception of professional support by the parents especially in the emotional area (Ragnarsdóttir & Svavarsdottir, 2014).

This case study was able to elaborate that the woman was perceiving more ease by modeling burdens and suffering. The burdens and suffering were not resolved, but new perspectives were developed. In the study by Robinson (1998), which was conducted in a similar setting to this study, the effect of a few therapeutic conversations to the mothers’ suffering was partly due to the increased awareness of maternal stress among all family members. Persson and Benzein (2014), investigating therapeutic conversations with five families with an adult chronic ill family member, observed a conversation progression of narrating, listening, reconsidering, and developing an expanded consciousness toward more hope and improved family health, which was characterized by the interaction between counselor and family, but also among family members. In our study, however, the conversations took place between a single mother and a counselor. Nevertheless, the conversation focused on the extended family system, for example, with the help of the genogram-ecomap, which also seemed to support finding a more balanced family life.

The woman’s main concern was her own health, not that of her son, which caused her existential worries. She was concerned about her own health to be able to look after herself and her children. The results suggest that the high demands of being a single mother and a mother of a child with intellectual disability put additional strain on her pre-existing health problems. In the study by Kim et al. (2023), single parents mentioned physical and mental health problems that were experienced as particularly threatening to the care of the dependent child due to the lack of partnership. In this case study, the counselor was able to support the woman’s perceptions and beliefs regarding her health problems, discussed persistent overwhelm in developing new solutions, and encouraged formulating smaller goals, as recommended by Wright et al. (2021) to strengthen the cognitive family function domain. Schwing and Fryszer (2016) confirm that a realistic prospect of success of goals is a prerequisite for their effectiveness.

The woman’s health problems further exacerbated the financial burden and the associated existential worries of the woman. Previous studies have shown financial restrictions for families with children with disabilities due to the care required and additional costs incurred by the child (Mitterer et al., 2021; Roddy, 2022; Wondemu et al., 2022), which is even more pronounced for single mothers (Kim et al., 2023). In this case study, a further significant aggravation of the financial situation became apparent due to the woman’s own health problems.

Furthermore, the need for peace and quiet crystallized, whereby it is difficult to find a family balance between the individual needs of the family members and to organize everyday life. Roskam and Mikolajczak (2023) showed that both being a parent of a child with a disability and being a single parent of healthy children increased signs of burnout and worsened the balance between risks and resources, as compared with other parents. Both negative outcomes increased linearly when both constellations applied together (Roskam & Mikolajczak, 2023). Single parents with children with disabilities are therefore affected by a double vulnerability (Kim et al., 2023; Roskam & Mikolajczak, 2023). The doubly increased imbalance between risks and resources due to their situation and the doubly increased signs of burnout (Roskam & Mikolajczak, 2023) can explain the difficulty of this woman to organize external support despite her need for rest. According to a long-term study, the social support for mothers even decreased with the severity of the child’s disability, which in turn increased the mothers’ risk of depression and anxiety (Carlson & Miller, 2017). This indicates a negative spiral difficult to overcome with the therapeutic conversations being helpful to start taking control of change and thus supporting the behavioral family function domain (Shajani & Snell, 2023).

Regarding the questions of guilt and blame, the counselor offered the woman reinterpretations and reframing to transform her cognitive frame of reference with a positive connotation and to evaluate the urgently needed recovery breaks in a positive way. Issues of guilt contribute to chronic sorrow, especially in mothers (Coughlin & Sethares, 2017), which was also apparent in this case study. In a study of single mothers of children with autism spectrum disorders, respite breaks led to daily uplifts that in turn reduced depressive symptoms (Dyches et al., 2016). Thus, this simple reframing intervention supported both the woman’s scope of actions for improved health and for more rest, which in turn are prerequisites for more independence.

Moreover, a great suffering of the woman regarding political injustices was identified. The need to talk about it and to make the woman’s environment and the society aware of it became clear. However, the woman perceived the people’s lack of understanding and disinterest in political injustices in Switzerland leading to strong emotions and a confusion that resulted in a feeling of not belonging. Through allowing a moment of silence during the conversation, the counselor legitimized the woman’s feelings and perceptions on a nonverbal level and provided her with a sense of belonging helping to reduce her stress. According to Wright and Bell (2021), health and illness are understood in the physical, emotional, relational, and/or spiritual domains. Based on this case study, it could be argued that this understanding should be expanded to include the cultural dimension, as acculturation stress has an impact on mental health and needs more awareness in nursing care (Horne, 2024).

### Strengths and Limitations of the Study

The single-case study design proved to be suitable for gaining an in-depth understanding of the individual case in its complexity, especially with the family system focus (Schulz et al., 2021). Nevertheless, the results of the individual case may not be transferable for families in similar situations, since each family has individual characteristics and a different context. In addition, the conversations provided limited insight into the family history, the suffering, and the context from the woman’s perspective. The conversations were conducted by one counselor, who was an experienced advanced practice nurse, which may raise the question of potential differences to conversations conducted by other counselors, for example, regarding professional background, expertise, sympathies, or individual assumptions. The counselor conducting the conversations relied on established conversation models and a sound theoretical background to ensure a professional approach to the therapeutic conversations. In addition, each conversation was analyzed and reflected upon in detail by the Family Systems Care Unit team in the preliminary and evaluating sessions. This contributed to the effectiveness of the therapeutic conversations and the validity of the study results. Finally, the data collection of this study must be distinguished from a traditional research interview. The therapeutic conversations provided the woman a setting for benefiting directly from participation, which is an ethical strength of this study.

### Implications for Practice and Research

Health care and research on children with intellectual disability requires professionals who can respond to the complex needs of these children (Totsika et al., 2022). However, the challenges faced by the family in this case study go far beyond the complexity that other families face while raising a child with an intellectual disability. It is crucial, but not sufficient, to place the needs and challenges of the child at the center of health care and research. This study highlights that the use of a genogram-ecogram is highly valuable in clinical nursing practice, as it helps to capture the complexity of family situations and facilitates a deeper understanding of the values, beliefs, and relationships within the family.

To promote the feeling of more ease in burdened parents, particularly in a single mother with multiple burdens, deliberately and explicitly acknowledging their competencies, along with recognizing and commending their courage and strengths in navigating complex challenges, is helpful. Targeted normalization, such as by sharing personal experiences, can also be relieving, as this reassures parents that they are not alone in facing difficulties. In addition, validating feelings, experiences, and challenges, alongside to creative interventions like reframing, proves useful in alleviating distress caused by guilt and blame and in strengthening parental resilience. Moreover, the intentional use of silence and nonverbal communication can create space for reflection and foster a sense of belonging and acceptance.

Furthermore, there is a need for more easily accessible services, such as the Family Systems Care Unit, where challenges can effectively be addressed based on broad professional expertise. More generalist health care professionals who are not focused on one population but are able to respond to multilayered challenges viewing families as an entity are needed. In addition, health and social issues intersect in this case study, indicating the demand for more collaboration between social and health care services.

For future research, other families in similar situations should be included and compared in multiple case studies to identify common themes. More research is needed on the complex challenges of single parents of children with disabilities and the effects of family-systemic therapeutic conversations with individuals, especially with highly burdened single parents.
